# Comparative Proteomic Analysis of Peritoneal Dialysate from Chronic Glomerulonephritis Patients

**DOI:** 10.1155/2013/863860

**Published:** 2013-05-14

**Authors:** Hsin-Yi Wu, Alex Chien Hwa Liao, Chien-Cheng Huang, Pao-Chi Liao, Chih-Chiang Chien, Wei-Chih Kan, Hsien-Yi Wang

**Affiliations:** ^1^Institute of Chemistry, Academia Sinica, Taipei 115, Taiwan; ^2^Division of Urology, Department of Surgery, Chi-Mei Medical Center, 901 Chung-Hwa Road, Yung-Kang District, Tainan City 710, Taiwan; ^3^Department of Senior Citizen Service Management, Chia Nan University of Pharmacy and Science, Tainan 717, Taiwan; ^4^Department of Emergency Medicine, Chi-Mei Medical Center, 901 Chung-Hwa Road, Yung-Kang District, Tainan 710, Taiwan; ^5^Department of Leisure, Recreation and Tourism Management, Southern Taiwan University of Science and Technology, Tainan 710, Taiwan; ^6^Department of Environmental and Occupational Health, National Cheng Kung University, Tainan 710, Taiwan; ^7^Division of Nephrology, Department of Internal Medicine, Chi Mei Medical Center, 901 Chung-Hwa Road, Yung-Kang District, Tainan 710, Taiwan; ^8^Department of Food Nutrition, Chung Hwa University of Medical Technology, Tainan 717, Taiwan; ^9^Department of Medical Laboratory Science and Biotechnology, Chung Hwa University of Medical Technology, Tainan 717, Taiwan; ^10^Department of Sport Management, College of Leisure and Recreation Management, Chia Nan University of Pharmacy and Science, Tainan 717, Taiwan

## Abstract

Peritoneal dialysis (PD) frequently contributes to peritoneal damage which cannot be easily identified without invasive techniques, implying the urgent need for biomarkers and revealing mechanisms. Chronic glomerulonephritis (CGN) is one of the leading causes of receiving dialysis treatment. Here, we attempted to analyze the peritoneal dialysate collected from CGN patients when they receive continuous ambulatory peritoneal dialysis (CAPD) treatment for the first time and after a year to reveal the protein changes that resulted from PD. Proteins were displayed by two-dimensional gel electrophoresis (2DE). Altered gel spots were digested followed by liquid chromatography-tandem mass spectrometry (LC-MS/MS) analysis for protein identification. Eight proteins were found to have differential expression levels between two groups. Their differential expressions were validated by Western blots in other sets of peritoneal dialysates. Proteins identified with higher levels in the first-time dialysate suggested their dominant appearance in CGN patients, while those that showed higher levels in peritoneal dialysate collected after one year may result from initial peritoneal inflammation or changes in the permeability of the peritoneum to middle-sized proteins. All the identified proteins may provide a perceptiveness of peritoneal changes caused by PD and may function as potential biomarkers or drug targets.

## 1. Introduction

Peritoneal dialysis (PD) is a widely used method of renal replacement therapy which also can lead to pathologic damage of the peritoneum and patient fatality. One potential mechanism is glucotoxicity to the peritoneal membrane [1]. However, it is not possible to identify peritoneal change without invasive techniques up to now; therefore, biomarkers that can be used for recognizing initial damages caused by PD are of urgent need. Nevertheless, insufficient information about proteins that are changed during peritoneal dialysis or between different kinds of diseases makes pathogenic mechanism unclear. The studying of fluid from dialysis may reveal potential markers or shed light on the mechanisms of peritonitis.

In recent decades, analyses of peritoneal dialysate by proteomic approaches have opened up progresses in this field. A descriptive study was performed on the dialysate of nine paediatric PD children patients by SDS-PAGE [2]. A total of 189 proteins, involved in acute phase proteins, complement factors, hormones, coagulation factors, and apolipoproteins, were identified. Evidence shows that daintain/allograft inflammatory factor-1 (AIF-1) increases in the first spent dialysate from patients newly receiving continuous ambulatory peritoneal dialysis (CAPD), suggestion a potential marker of CAPD peritonitis [3]. Lin et al. have compared the protein profile of peritoneal dialysate from 16 patients with and without peritonitis [4] by two-dimensional gel electrophoresis (2DE), which identified *β*2-microglobulin as potential biomarker for CAPD peritonitis. Tyan et al. also analyzed 12 peritoneal dialysate collected from patients before and after peritonitis by 2DE and identified 10 proteins that were significantly differentially expressed between two groups [5]. A proteomic analysis was conducted on peritoneal dialysate from CAPD patients whose peritoneal membranes are of high (H), high average (HA), low average (LA), and low (L) transport rates [6]. Five proteins, identified as serum albumin in a complex with myristic acid and triiodobenzoic acid, *α*1-antitrypsin, complement component C4A, immunoglobulin *κ* light chain, and apolipoprotein A-I, were found to show differed levels among groups. Another laboratory works on analyzing the protein composition of peritoneal dialysate from patients receiving peritoneal dialysis with different concentration of glucose [7]. Four proteins, alpha-1-antitrypsin, fibrinogen beta chain, transthyretin, and apolipoprotein A-IV, were found to be underexpressed in the highest osmolar solution. The result provides potential targets for future therapeutic implementation in preventing inflammatory processes induced by the exposure to dialysis solutions. Besides, Wang et al. have compared the protein profile of diabetic peritoneal dialysate versus normal peritoneal fluid [8]. According to the western confirmation results, vitamin D-binding protein, haptoglobin, and *α*-2-microglobulin showed higher levels in the DM samples, while complement C4-A and IGK@ protein were of lower levels compared to the control samples.

Chronic glomerulonephritis (CGN), nephroangiosclerosis, and diabetes were identified as the most common causes of chronic renal failure [9, 10]. CGN is characterized by slowly inflammatory changes in glomerular capillaries generally leading to irreversible renal failure. Here, we intend to analyze the peritoneal dialysate collected from CGN patients when they receive CAPD treatment for the first time and after a year to point out the protein changes due to CGN-induced accumulation of uremic toxins or the inflammation caused by the oxidative stress. The altered protein targets may shed light to the mechanism of peritoneal change and function as clues to initial damage of peritoneal membrane. 

## 2. Material and Methods

### 2.1. Patient Sample Preparation

Ten CGN patients were recruited in this study. Their peritoneal dialysates were collected first time at their early stage of receiving maintenance dialysis treatment (E1–10). After one year of CAPD treatment, their peritoneal dialysates were collected again to present the middle stage of treatment (M1–10). The clinical data of these patients were provided in Supplementary Table A (see Supplementary Material available online at http://dx.doi.org/10.1155/2013/863860). This study was approved by the Institutional Research Board and carried out according to the Helsinki Declaration Principles. All participating subjects have provided written informed consents. Each sample was centrifuged at 1000 ×g at 4°C for 10 min, and the supernatants were stored at −20°C until further use. An aliquot of each sample was used to measure protein concentration.

### 2.2. Protein Precipitation

Peritoneal dialysates from seven of the ten patients were used for protein profiling by two-dimensional gel electrophoresis (2DE) analysis, while thoes from the other three patients were used for further validation of the altered proteins by Western blotting analysis. Peritoneal dialysates collected from two stages, E1–E7 and M1–M7 (each containing 140 *μ*g of protein), were run individually, and triplicate analyses were performed for each sample. Trichloroacetic acid in cold acetone (1 : 9) containing 0.1% dithiothreitol was added to each sample and then was placed at −20°C overnight to facilitate protein precipitation. Samples were next centrifuged at 18000 g for 30 min to collect the pellets. After three times of washing with acetone (−20°C) containing 0.1% dithiothreitol, the pellets were left air dried. Each pellet was dissolved in a 250 *μ*L rehydration solution (7 M urea, 2 M thiourea, 4% CHAPS, 2% ampholytes, and 120 mM dithiothreitol) and carefully sonicated for 60 min.

### 2.3. Two-Dimensional Gel Electrophoresis (2DE) and Image Analysis

Protein samples were loaded onto immobilized pH gradient (IPG) gel strips for the first-dimensional gel electrophoresis. To minimize evaporation and urea crystallization, IPG cover oil (0.8 mL) was applied on top of each gel strip. Isoelectric focusing (IEF) was run following a stepwise incremental voltage program on an IPGphor Isoelectric Focusing System (GE Healthcare Life Sciences, Taipei, Taiwan). Samples were run under the following settings: 30 V for 16 h, 500 V for 1 h, 500 V for 1 h, and 8000 V for 4 h. After IEF, the strips were first placed in equilibration buffer with 1% dithiothreitol w/v for 15 min followed by in 2.5% w/v iodoacetamide for another 15 min. The IPG strip was placed on a 1.0 mm thick 12% polyacrylamide gel and was run at 300 V for 4-5 h for the second-dimensional gel electrophoresis. The visualization of the gels was achieved by using silver staining. The stained gels were scanned at a resolution of 300 dpi using an ImageScanner operated by LabScan 3.00 software (GE Healthcare Life Sciences). Image spots were detected and matched by using ImageMaster 2D software (GE Healthcare). The spot detection was manually checked. Each spot intensity was normalized by the total intensity volume of all spots detected on the gel. To identify the differentially expressed protein spots during the CAPD treatment, spot intensities of the sample from middle-stage groups were compared to those from the early-stage groups. Ratios were statistically analyzed using Student's *t*-test, and a *P* value <0.05 was considered significant. Protein spots with altered expression were excised from the gels and subjected to protein identification.

### 2.4. In-Gel Digestion

The protein spots were washed with 25 mM ammonium bicarbonate and 50% acetonitrile/25 mM ammonium bicarbonate. After being washed three more times with 25 mM ammonium bicarbonate and 50% acetonitrile/25 mM ammonium bicarbonate, the gel fragments were placed at 56°C for 1 h in a solution containing 10 mM dithiothreitol, 55 mM iodoacetamide, and 25 mM ammonium bicarbonate for protein reduction and alkylation. Gel fragments were added with 10 *μ*L of a 0.1-*μ*g/*μ*L modified trypsin digestion buffer in 25 mM ammonium bicarbonate and were incubated at 37°C overnight. After tryptic digestion, the supernatants were transferred to Eppendorf tubes, and the remaining peptides were further extracted from the gel pieces by incubation with a solution of 50% acetonitrile and 5% formic acid.

### 2.5. Protein Identification Using Liquid Chromatography-Mass Spectrometry and Database Searching

Liquid chromatography-tandem mass spectrometry (LC-MS/MS) was used for protein identification. Tryptic peptides were fractionated in a C18 microcapillary column (75 *μ*m i.d. × 15 cm) at a flow rate of 200 nL/min with a nano-HPLC system (LC Packings Nederlands B.V., Amsterdam, The Netherlands). A 40 min gradient from 0 to 60% buffer B (80% acetonitrile, 0.1% formic acid) was used to elute the peptides. The eluate was electrosprayed into an ion trap mass spectrometer (LCQ DECA XP Plus; Thermo Electron Corp., San Jose, CA, USA) using a distal 1.3 kV spraying voltage. Each cycle of one full scan mass spectrum (m/z 150–2000) was followed by three data-dependent tandem mass spectra on the three most intense peaks. For mass spectrometry data analysis, Xcalibur BioWorks 3.3 software (Thermo Fisher Scientific, Waltham, MA, USA) was used to generate peak lists. The resulting DTA files were merged and searched against Swiss-Prot human protein sequence database by using Mascot software (Version 2.2.1, Matrix Science, London, UK). The search parameters were set as follows. A maximum of two missed cleavage sites were allowed. Variable modifications were set on cysteines (carboxyamidomethylation), methionine (oxidation), and asparagine and glutamine (deamidation). The mass tolerance of both the precursor peptide ions and the fragment ion was set to 0.5 Da. For peptide identification, the peptides identified with expected values <0.05 were acceptable. Proteins identified with a Mascot score above the significant hit threshold (*P* < 0.05) and more than two rank 1 peptides were considered significant.

### 2.6. Western Blotting

Samples, each contained 25 *μ*g of proteins, were loaded in 1.0 mm × 10-well gradient gels (NuPAGE Bis-Tris, 4% to 12%) purchased from Invitrogen (Carlsbad, CA, USA). Proteins were resolved in the gel and transferred onto nitrocellulose membranes. The membranes were blocked in 5% nonfat milk in Tris-buffered saline with Tween 20 (TBST, containing 3.0 g/L Tris, 14.4 g/L glycine, and 0.5% Tween 20 with pH adjusted to 8.3) at room temperature for 1 h. The membranes were then washed three times with TBST and probed with antibodies at 4°C overnight. The primary antibodies and corresponding concentrations used were as follows: anti-Ig mu chain C region (1 : 2000) (MyBioSource, Vancouver, BC, Canada); antifibrinogen gamma chain (1 : 1000) (Abcam, Cambridge, UK); anti-C-reactive protein (1 : 500) (Santa Cruz Biotechnology, Inc., Santa Cruz, CA, USA); anti-Ig delta chain C region (1 : 500) (Santa Cruz Biotechnology, Inc.); anti-alpha-1-antitrypsin (1 : 1000) (Abcam); anti-histidine-rich glycoprotein (1 : 1000) (R&D Systems, Minneapolis, MN, USA); antiapolipoprotein A-I (1 : 5000) (Epitomics, Burlingame, CA, USA); and antiserum amyloid P (1 : 5000) (Abcam). After being washed three times with TBST and incubated with horseradish peroxidase-conjugated secondary antibodies (1 : 2000–1 : 5000) at room temperature for 1 h, membranes were then washed with TBST for three more times and then developed with enhanced chemiluminescence detection. The volume densitometry of each band was determined using ImageMaster 2D software.

## 3. Results

### 3.1. Analysis of Individual Peritoneal Dialysate Samples Using 2DE

To reveal the protein profile change due to CAPD treatment, 2DE was used in this study. Peritoneal dialysates were collected from seven CGN patients at the beginning of receiving CAPD treatment, which was used to present the early stage of peritoneal dialysate sample (E1–E7). These patients were treated with CAPD, and the middle-stage peritoneal dialysate samples were collected after a year. Each sample was analyzed individually and repeated three times.

**Figure 1:**
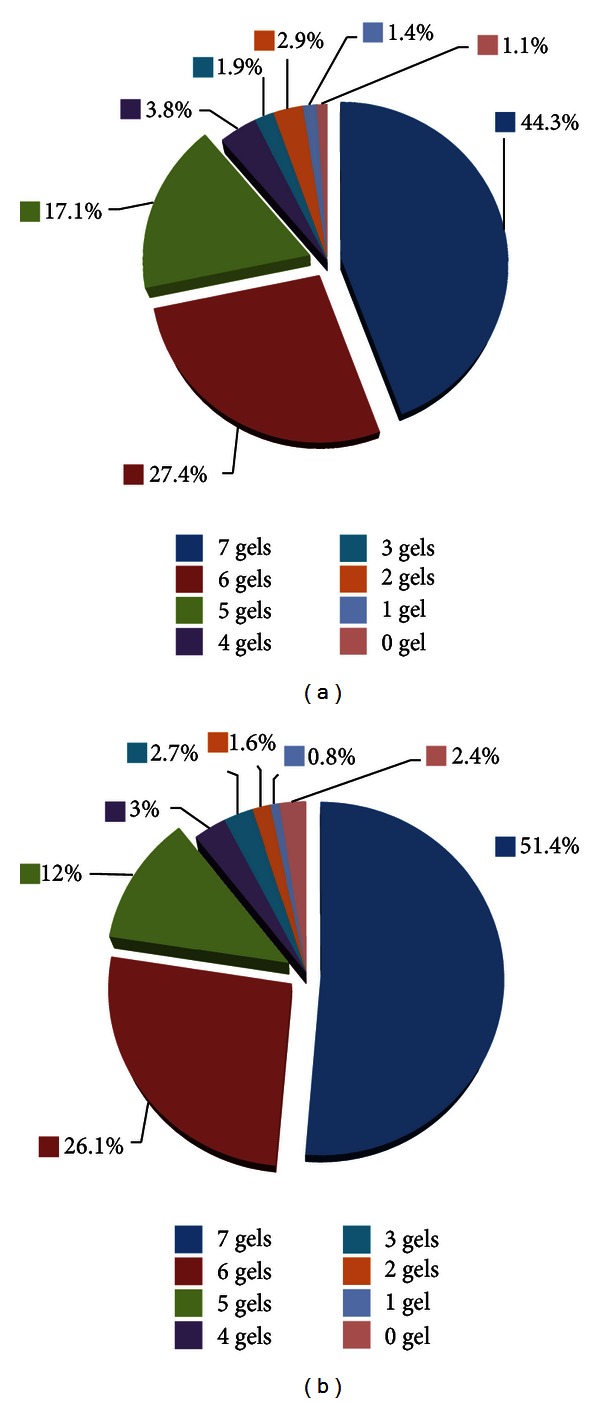
Evaluation of the individual difference within each of the 7 peritoneal dialysates collected from (a) early stage and (b) middle stage. A total of 386 spots were detected in the master gel of those 14 gels. Among the E1–E7 gels, 163 (44.3%), 101 (27.4%), 63 (17.1%), 14 (3.8%), 7 (1.9%), 11 (2.9%), 5 (1.4%), and 4 (1.1%) spots were detected in 7, 6, 5, 4, 3, 2, 1, and 0 of the early-stage gels, respectively. Among the M1–M7 gels, 189 (51.4%), 96 (26.1%), 44 (12.0%), 11 (3.0%), 10 (2.7%), 6 (1.6%), 5 (0.8%), and 9 (2.4%) spots were detected in 7, 6, 5, 4, 3, 2, 1, and 0 of the middle-stage gels, respectively.

To evaluate the reproducibility of the 2DE gels, twenty-one gels were subjected to image analysis. We found that approximately 95% of the spots can be detected within each triplicate experiment. Since the discrepancy of the gel images was determined by the reproducibility of the presence of each protein spots, here we counted the number of gels in which each spot appeared to evaluate the variance within each sample. Owing to the high similarity between the triplicate gels, a submaster gel can be generated from each patient sample at two different states. By combining all submaster gels (14 images), a master gel containing 368 spots was obtained. Among them, 163 (44.3%), 101 (27.4%), and 63 (17.1%) spots can be detected in 7, 6, and 5 gels of the early-stage gels (E1–E7), respectively, consisting of 88.8% of all the datable spots (Figure 1(a)). Besides, the remaining 14 (3.8%), 7 (1.9%), 11 (2.9%), 5 (1.4%), and 4 (1.1%) spots were detected in 4, 3, 2, 1, and 0 of the E1–E7 gels, respectively. As for the M1–M7 groups, as depicted in Figure 1(b), 9.5% of the 368 spots, 189 (51.4%), 96 (26.1%), and 44 (12.0%) spots, were found in 7, 6, and 5 gels, respectively. The other 11 (3.0%), 10 (2.7%), 6 (1.6%), and 5 (0.8%) spots were detected in 4, 3, 2, and 1 of the middle-stage gels, respectively, while 9 (2.4%) spots were absent in all gels. The absence of spots in one group suggests that differences between early- and middle-stage peritoneal dialysate exist. Although several proteins showed differential expression, no consistent, statistically valid (*P* < 0.05) differences between the E1–E7 and M1–M7 groups were detected.

**Figure 2:**
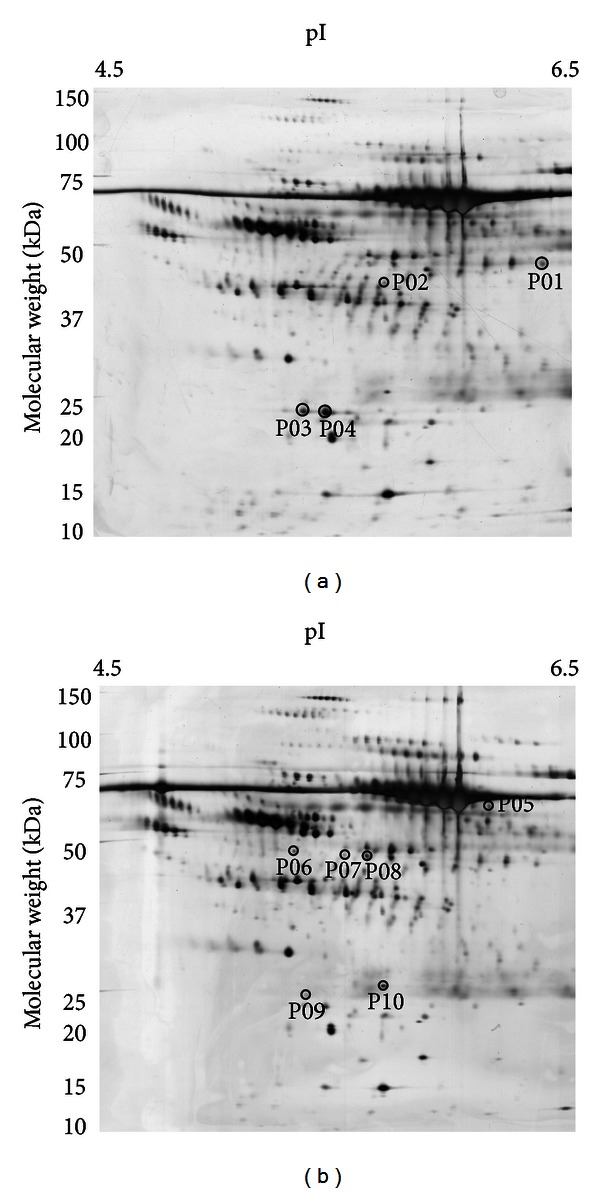
Representative 2DE gels of the (a) early-stage and (b) middle-stage samples. Each sample containing 140 *μ*g was analyzed separately by 2DE (pH 4–7). The analysis of each sample was repeated three times. About 300 protein spots were detected on each gel, and a total of 386 protein spots were contained in the final master gel. Comparing between the two groups, 10 proteins were found to have differential levels, of which, 4 spots (P01–P04) had higher levels in the early-stage samples (pointed out by arrows in panel (a)), while 6 spots (P05–P10) showed higher levels in the middle-stage ones (indicated by arrows in panel (b)).

After evaluation of the similarity between samples, E1–E7 and M1–M7 (each with triplication) consisting of 42 gels were comparatively analyzed to identify CAPD induced protein change. Gel spots were analyzed by image analysis software for spot detection, spot matching, intensity normalization, and result output.  Figure 2 was of two representative gels of early-stage (Figure 2(a)) and middle-stage peritoneal dialysate samples. The differential change of each spot was estimated by Student's *t*-test. Spots with expression levels that varied between E1–E7 and M1–M7 samples with a *P* value <0.05 were considered significant. A total of 4 spots (Figure 2(a)) had significantly higher protein expression levels in the early-stage peritoneal dialysate than that in the middle stage. Six spots (Figure 2(b)) showed significantly higher protein expression levels in the peritoneal dialysate after one year of CAPD treatment.

### 3.2. Protein Identification by Liquid Chromatography-Tandem Mass Spectrometry (LC-MS/MS) Analysis

To identify these 10 altered protein spots, P01–P10 gel spots were excised, enzymatically digested, and subjected to LC-MS/MS analysis. Each spot was identified by searching the raw data against human protein database. Detailed information about each spot was listed in Table 1. All the proteins were identified with at least three peptides, suggesting that protein identification is reasonably accurate. The 10 spots were found to represent 8 proteins. Among them, 4 protein spots, showing higher levels in the early-stage sample, P01, P02, and P03/P04, were identified as Ig mu chain C region, fibrinogen gamma chain, and C-reactive protein, respectively. Other 6 spots, Ig delta chain C region (P05), alpha-1-antitrypsin (P06), histidine-rich glycoprotein (P07/P08), apolipoprotein A-I (P09), and serum amyloid P-component (P10), were identified with higher level in middle-stage samples. As listed in Table 1, for most proteins, the experimental molecular weight (MW) measured by 2D PAGE corresponded to the theoretical MW annotated in protein sequence database. For Ig mu chain C region (P01) and Ig delta chain C region (P05), their observed MW was higher than the theoretical value, which may have resulted from the posttranslational modification (e.g., glycosylation). The experimental isoelectric point (pI) of most proteins matched the theoretical value found in the database. It has been known that protein modifications also can alter pI. Therefore, we hypothesized that inconsistencies between observed and predicted pI (e.g., P06 and P07/P08) may indicate the presence of protein modification or processing. The subcellular location and protein functions were analyzed through gene ontology and summarized in Table 2.

### 3.3. Protein-Protein Interaction Network Analysis

**Figure 3:**
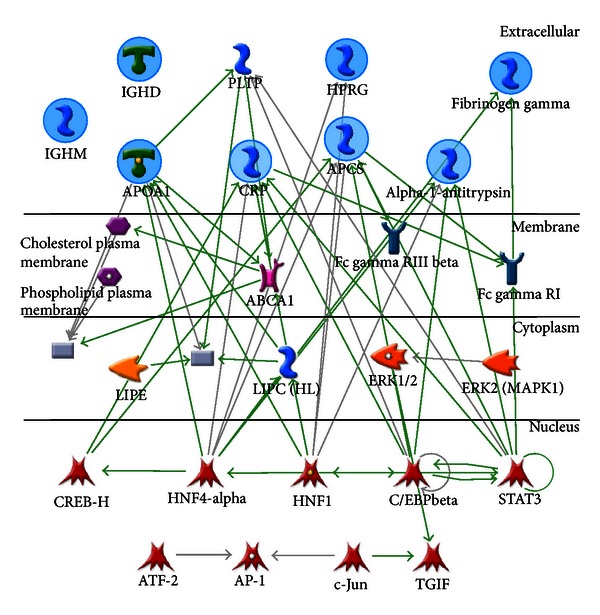
Interaction network analysis of 8 proteins that showed altered levels between early- and middle-stage groups. Proteins identified in this study, alpha-1-antitrypsin, serum amyloid P-component (APCS), C-reactive protein (CRP), fibrinogen gamma, apolipoprotein A-I (APOA1), histidine-rich glycoprotein (HPRG), Ig delta chain C region (IGHD), and Ig mu chain C region (IGHM), were marked with a circle. Full protein names for the abbreviation and legend for molecules used in MetaCore networks are provided in Supplementary Table  B and Supplementary Figure  A.

Proteins rarely act alone. Most of the time, they team up to carry out biological functions. Mapping of protein-protein interaction can help unravel the complicated molecular relationships in living systems. Here, in order to elucidate the main molecular interactions and biological connections, a protein-protein interaction network was constructed based on the 8 altered proteins by MetaCore (GeneGo, Inc., St Joseph, MI, USA). (illustrated in Figure 3). The full name of the abbreviation was provided in Supplementary Table  B. The indication of the shape and color of the molecule was depicted in Supplementary Figure  A. The protein molecules in the interaction network were laid out according to their cellular localizations. The network consisted of 25 proteins, among which, 6 proteins, alpha-1-antitrypsin, serum amyloid P-component (APCS), C-reactive protein (CRP), fibrinogen gamma chain, apolipoprotein A-I (HDL), histidine-rich glycoprotein (HPRG) were identified in our study (indicated with circles). These 6 proteins were involved in a network that contained receptor ligand (e.g., HDL), binding protein (e.g., phospholipid transfer protein, PLTR), receptor (e.g., high affinity immunoglobulin gamma Fc receptor I, Fc gamma RI), ion channel (e.g., ATP-binding cassette subfamily A member 1, ABCA1), kinase/phosphatase (e.g., ERK1/2), phospholipase (e.g., endothelial lipase, LIPE), and various transcription factors (e.g., c-Jun).

### 3.4. Validation of Protein Expression by Western Blotting

**Figure 4:**
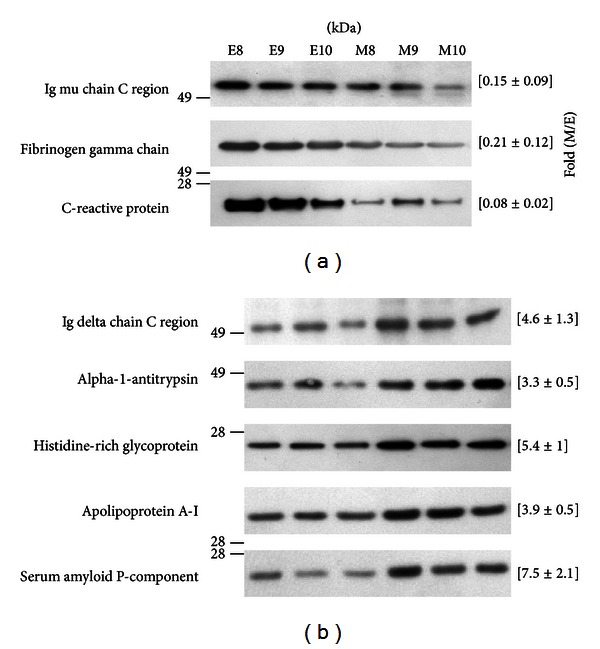
Western blotting of the identified proteins in peritoneal dialysates collected from other three CGN patients at their first time (E08–E10) and after a year (M08–M10) of receiving CAPD treatment to further validate these protein candidates. The mean band density of E08–E10 and M08–M10 was calculated and used to obtain the fold change between the two groups (M/E). The fold is indicated in the bracket to the right of each set of bands. Western blotting confirmed that (a) Ig mu chain C region, fibrinogen gamma, and C-reactive protein show higher expression levels in the early-stage samples than in the middle-stage samples, while (b) Ig delta chain C region, alpha-1-antitrypsin, histidine-rich glycoprotein, apolipoprotein A-I, and serum amyloid P-component were observed to have higher levels in the dialysate after one year of CAPD treatment.

To further confirm the results of 2DE analysis, Western blotting was used to evaluate the expression levels of altered proteins in peritoneal dialysates from three other CGN patients (E8–E10 versus M8–M10). The fold change in the expression of each identified protein was obtained by dividing the mean band density of middle-stage sample by that of the early-stage samples (M/E). The expression levels of the three proteins, which were considered with higher levels in early-stage samples by 2DE, were confirmed by using Western blotting in three separate samples. As shown in Figure 4(a), Ig mu chain C region (P01, fold change = 0.15), fibrinogen gamma chain (P02, fold change = 0.21), and C-reactive protein (P03/P04, fold change = 0.08) were detected. As for those proteins that were found to have higher levels in middle-stage samples, the values of fold change evaluated by Western blotting were 4.6, 3.3, 5.4, 3.9, and 7.5 for Ig delta chain C region (P05), alpha-1-antitrypsin (P06), histidine-rich glycoprotein (P07/P08), apolipoprotein A-I (P09), and serum amyloid P-component (P10), respectively (Figure 4(b)). These results are consistent with that from 2DE analysis.

## 4. Discussion

In order to comprehensively realize the protein group involved, a protein network analysis of the 8 altered proteins was done to deduce their interacting proteins. Six of the 8 identified proteins can be linked through protein-protein interaction, suggesting their close association in the system. Some interesting proteins were also recruited in the network and were found to be related to the process of inflammation or immune response. ABCA1 deficiency can lead to impaired cholesterol efflux to HDL or apolipoprotein A-I (APOA1), decreased apoE secretion, and increased secretion of inflammatory cytokines and chemokines [11]. In the deletion of Fc gamma RI, antibody responses were elevated, implying its role as a control point in the development of immune responses [12]. The copy number variation of the FCGR3B gene (Fc gamma RIII beta) is found to be associated with immune complex clearance [13]. The downstream moles in cytoplasm and nucleus, such as ERK1/2, c-Jun, AP-1, ATF-2, and STAT3, have been known to participate in inflammatory response [14–17]. Those interacting proteins indicate that these differentially expressed proteins are involved in peritoneal inflammation.

We speculated that proteins identified with higher levels in the first-time dialysate suggested their prevailing expression in CGN patients. Ig mu chain C region (P01) was identified with higher levels in the initial peritoneal dialysate. According to a previous work, patients with primary glomerulonephritis showed IgM as dominant molecules in glomerular by immunofluorescence [18]. A recent work found that mouse with Ig mu chain C region gene knockout was devoid from the glomerular damage, implying the requirement of Ig mu chain C region in the development of glomerulonephritis. Several lines of evidence supporting the correlation between fibrinogen and glomerulonephritis have been obtained [19]. Our finding of the appearance of fibrinogen gamma chain (P02) in the peritoneal dialysate from CGN patients is consistent with those previous works. The expression of human C-reactive protein (P03/P04) in the lupus-prone NZB/NZW F1 mice has delayed the initiation of glomerulonephritis and death [20], suggesting that high level of C-reactive protein may present a self-protective process against glomerulonephritis.

Proteins that have higher levels in peritoneal dialysate after one year of CAPD treatment may shed light on the mechanism or potential marker for initial peritoneal damage. The main role of Ig delta chain C region (P05) in immune system has been believed to be B-cell receptor. The expression of Ig delta chain C region is required in mature B cells and a key modulator of the humoral immune response (reviewed in [21]). It has been known that alpha-1-antitrypsin (P06) is detectable in peritoneal dialysate of peritonitis-free patients. In peritonitis-free patients, alpha-1-antitrypsin prevents the proteolytic action and cell activation, leading to platelet activating factor synthesis. However, the inactivation of its function by oxidants resulted in proteolytic injury and unrestrained synthesis of inflammatory mediators during peritonitis [22]. Histidine-rich glycoprotein (P07/P08) has been shown to bind heparin [23], plasminogen [24], and fibrinogen [25] and to regulate aspects of the immune system [26, 27]. Histidine-rich glycoprotein is an important regulator of immune complexes uptake by monocytes [28]. Levels of plasma histidine-rich glycoprotein have been found to decrease during acute states of disease such as advanced liver cirrhosis [29], AIDS, renal disease, asthma, and pulmonary disease [30]. Histidine-rich glycoprotein can interact with different cell surface receptors and bind a wide range of ligands, and it has been involved in the regulation of numerous biological functions, particularly angiogenesis, immune complex clearance, and coagulation (reviewed in [31]). Apolipoprotein A-I (P09) was higher in middle-stage than in early-stage samples, which is consistent with previous studies in which patients with high transport rates in peritoneum also had higher apolipoprotein A-I in dialysate when compared to those with low transport rates [6]. Apolipoprotein A-I is the major component of HDL and functions as an acceptor for the sequential transfer of phospholipids and free cholesterol from peripheral tissue. It also facilitates the transport of cholesterol to the liver and other tissue for excretion and steroidogenesis. [32]. The loss of apolipoprotein A-I in peritoneal dialysate effluents led to the decrease of apolipoprotein A-I and HDL in plasma [33], which implied the reason why patients with high solute transport rates are prone to develop atherosclerosis [34]. Serum amyloid P component (P10) is an acute phase protein, structurally related to C-reactive protein, which is synthesized in response to the proinflammatory cytokines early in the inflammatory. Serum amyloid P component interacts with inflammatory and complement factors, acting as an opsonin that reacts with nuclear autoantigens in systemic autoimmunity [35]. Mice with targeted deletion of serum amyloid P component gene lead to the development of immune complex glomerulonephritis. In adult female mouse, homozygous mutant mouse with serum amyloid P component gene knockout increases glomerulonephritis [36]. Both studies designated that this protein is crucial for the development of glomerulonephritis. The observation of those proteins showing differential levels at two stages in peritoneal dialysate may provide novel aspects for the study initial peritoneal change related to peritoneal dialysis. These proteins may help elucidate the mechanism of inflammation, peritoneal structural change, kidney damage, atherosclerosis, angiogenesis, and fibrosis related to dialysis.

This study has its limitations. It is very difficult to identify peritoneal damage unless invasive methods are used. Therefore, we cannot define the condition of peritoneal change, but we can assume that the peritoneum has slightly changed after the long-term CAPD treatment. This is a major obstacle in our experimental research. In addition, the small number of patients may contribute to the limited biological and clinical relevance of our findings. To address this issue, the differentially expressed proteins were further validated in another set of 3 CGN patients. This work has provided potential targets and mechanisms of the change due to peritoneal dialysis processes, which have been confirmed by mass spectrometry data and Western blotting. Before being applied clinically, further experiments are considered necessary to evaluate those targets in a sufficient number of participants.

In conclusion, to our knowledge, this is the first study to show differential proteomic profiling between peritoneal dialysates from CGN patients at the early and middle stage of CAPD treatment. The identified proteins may provide clues to the PD-induced loss of proteins from the peritoneum and assist the identification of potential biomarkers for noninvasive monitoring of peritoneal damage. Further studies of these potential proteins are needed for accessing their roles in both basic research and clinical monitoring.

## Supplementary Material

The clinical data of the 10 patients, used for 2DE experiment (P1-P7) and Western blotting (P8-P10), at their early and middle stage of PD were provided in Supplementary Table A. The full names of the proteins shown in Figure 3 were provided in Supplementary Table B while the legend of the molecules used in Figure 3 was provided in Supplementary Figure A.

## Figures and Tables

**Table 1:** Identification of 10 altered spots, of which 4 showed higher levels in the early-stage PD samples, while 6 had higher levels in the middle stage.

Spot number^a^	Protein name	Swiss-Prot accession no.	Mr (E/T)^b^ (kDa)	pI (E/T)^c^	No. of identified peptides	Sequence coverage (%)	Mascot score	Fold change ± SD^d^	Alteration (M/E)
P01	Ig mu chain C region	P01871	68/49	6.5/6.4	5	10.2	215	0.34 ± 0.20	Down
P02	Fibrinogen gamma chain	P02679	45/51	5.7/5.4	6	11.8	258	0.09 ± 0.02	Down
P03	C-reactive protein	P02741	25/25	5.5/5.5	4	16.2	145	0.13 ± 0.06	Down
P04	C-reactive protein	P02741	25/25	5.4/5.5	5	20.1	156	0.21 ± 0.09	Down
P05	Ig delta chain C region	P01880	70/47	6.2/6.8	3	6.3	124	2.91 ± 0.65	Up
P06	Alpha-1-antitrypsin	P01009	46/47	4.7/5.4	6	12.8	301	55.51 ± 13.71	Up
P07	Histidine-rich glycoprotein	P04196	53/59	5.6/7.0	4	6.7	186	8.13 ± 1.20	Up
P08	Histidine-rich glycoprotein	P04196	53/59	5.7/7.0	5	8.4	211	13.11 ± 3.05	Up
P09	Apolipoprotein A-I	P02647	25/31	5.3/5.6	4	12.9	201	23.67 ± 2.89	Up
P10	Serum amyloid P-component	P02743	27/25	5.7/6.1	6	24.5	298	5.78 ± 1.09	Up

^a^The spot numbers are designated in Figures 2(a) and 2(b).

^b^Mr (E/T): experimental observation of apparent molecular weight in 2-DE/theoretical molecular weight calculated from protein sequence database.

^c^pI (E/T): experimental observation of pI in 2-DE/theoretical pI calculated from protein sequence database.

^d^Fold change and standard deviation calculated from the protein spot intensity in middle-stage samples (M) versus that in early-stage samples (E).

**Table 2:** Gene ontology analysis of 8 differentially expressed proteins.

Spot number^a^	Protein name	Subcellular location	Biological process	Molecular function
P01	Ig mu chain C region	Plasma membrane	Immune response	Antigen binding

P02	Fibrinogen gamma chain	Extracellular space	Platelet activation, platelet degranulation, protein polymerization, response to calcium ion, and signal transduction	Cell surface binding and metal ion binding

P03/P04	C-reactive protein	Extracellular space	Acute-phase response, complement activation, regulation of lipid storage, regulation of macrophage, opsonization, protein polymerization, response to hypoxia, and response to lead ion	Cell surface binding, cholesterol binding, choline binding, low-density lipoprotein particle binding, and metal ion binding

P05	Ig delta chain C region	Extracellular space	Immune response	Antigen binding

P06	Alpha-1-antitrypsin	Extracellular space	Acute-phase response, platelet activation, platelet degranulation, and regulation of proteolysis	Serine-type endopeptidase inhibitor activity

P07/P08	Histidine-rich glycoprotein	Extracellular space	Angiogenesis, chemotaxis, regulation of angiogenesis, regulation of cell adhesion, regulation of cell growth and proliferation, regulation of fibrinolysis, platelet activation and degranulation, regulation of immune, response to tumor cell, and regulation of gene expression	Cell surface binding, cysteine-type endopeptidase inhibitor activity, heme binding, heparan sulfate proteoglycan binding, heparin binding, immunoglobulin binding, metal ion binding, serine-type endopeptidase inhibitor activity, and zinc ion binding

P09	Apolipoprotein A-I	Extracellular space	Cholesterol metabolism, lipid metabolism, lipid transport, steroid metabolism, sterol metabolism, and transport	Cell surface binding, heme binding, heparin binding, immunoglobulin binding, metal ion binding, and zinc ion binding

P10	Serum amyloid P-component	Extracellular space	Acute-phase response and protein folding	Metal ion binding and unfolded protein binding
