# Supplementary material for: Comparative Proteomic Analysis of Peritoneal Dialysate from Chronic Glomerulonephritis Patients
Source: Biomed Res Int. 2013 May 14;2013:863860. doi: 10.1155/2013/863860 (PMC3666394; doi:10.1155/2013/863860)
Supplement: Supplementary file 1 — The clinical data of the 10 patients, used for 2DE experiment (P1-P7) and Western blotting (P8-P10), at their early and middle stage of PD were provided in Supplementary Table A. The full names of the proteins shown in Figure 3 were provided in Supplementary Table B while the legend of the molecules used in Figure 3 was provided in Supplementary Figure A. [file 863860.f1.pdf]

Supplementary Table A. Clinical data of the 10 patients recruited in this study.

(1) Early Stage

	**Reference range**	**P1**	**P2**	**P3**	**P4**	**P5**	**P6**	**P7**	**P8**	**P9**	**P10**
Age (year)		58	77	32	47	56	46	30	46	54	63
Sex		M	M	M	F	F	F	M	M	F	F
Hemoglobin (g/dl)	11.6 – 14.8 (female) 13.5 – 17.5 (male)	8.7	10.6	10.9	8.3	11.3	9.2	11.0	12.6	13.9	11.8
Hematocrit (%)	34 – 44 (female) 40 – 49 (male)	27.6	31.8	32.5	24	34	28.1	33.2	37.3	26.4	31.6
White-cell count (per mm3)	3,200 – 9,200	7400	6100	4800	6200	9200	7400	8500	5200	4600	6400
Platelet count (mm3)	150,000 – 400,000	152000	337000	151000	222000	371000	348000	224000	167000	263000	356000
Blood urea nitrogen (mg/dl)	6 – 22	55	46	62	62	56	54	40	64	54	64
Creatinine (mg/dl)	0.6 – 1.3	9.6	13.6	11	12	11.9	9.5	5.3	8.3	10.2	9.8
Fasting sugar (mg/dl)	70 – 100	157	136	83	137	215	90	95	142	89	123
Sodium (mEq/l)	135 – 148	140.6	141.2	146.1	141	139.9	139.7	141.8	143.9	139.7	141.3
Potassium (mEq/l)	3.5 – 5.0	3.52	4.10	4.2	2.98	3.38	4.00	3.46	3.63	3.85	4.36

Aspartate aminotransferase (IU/l)	10 - 50	30	25	20	17	15	17	23	36	45	34
Alanine aminotransferase	10 – 50	38	23	9	13	13	13	28	23	24	16

(2) Middle Stage

	Reference range	P1	P2	P3	P4	P5	P6	P7	P8	P9	P10
Age (year)		58	77	32	47	56	46	30	46	54	63
Sex		M	M	M	F	F	F	M	M	F	F
Hemoglobin (g/dl)	11.6 – 14.8 (female) 13.5 – 17.5 (male)	8.7	10.8	9.5	9.3	10	8.4	11.4	12.7	13.3	12.2
Hematocrit (%)	34 – 44 (female) 40 – 49 (male)	27.6	32.5	28.6	27.6	29.5	26	33.9	36.8	28.1	30.4
White-cell count (per mm3)	3,200 – 9,200	6700	5900	7300	7600	10300	5400	7100	6300	5200	7300
Platelet count (mm3)	150,000 – 400,000	178000	315000	250000	261000	335000	304000	165000	214000	289000	378000
Albumin (g/dl)	3.8 – 5.3	3.8	3.9	3.8	3.9	3.8	3.2	4.0	4.1	4.9	3.4
Blood urea nitrogen (mg/dl)	6 – 22	58	68	59	59	40	54	57	67	55	69
Creatinine (mg/dl)	0.6 – 1.3	10.1	13.7	12	10.19	14.17	9.24	7.56	8.8	11.6	10.1
Fasting sugar	70 – 100	144	91	85	90	106	91	84	145	91	131

(mg/dl)											
Sodium (mEq/l)	135 – 148	140.2	139.6	148.1	141.8	141.2	138.2	144.1	141.2	141.7	145.2
Potassium (mEq/l)	3.5 – 5.0	3.7	4.20	3.69	3.68	4.86	5.01	3.83	4.25	3.27	4.83
Aspartate aminotransferase (IU/l)	10 – 50	27	19	17	15	23	15	21	32	41	33
Alanine aminotransferase (IU/l)	10 – 50	31	21	13	10	21	15	17	21	26	21
Calcium (mg/dl)	8.4 – 10.2	8.7	9.7	9.7	9.9	9.8	8.4	9.2	8.8	9.2	9.5
Phosphate (mg/dl)	2.3 – 4.7	4.2	6.5	4.9	4.1	7.3	6.0	4.2	5.2	6.7	4.9
Bilirubin, direct (mg/dl)	0 – 0.4	-	0.05	0.05	0.03	0.08	0.05	0.22	0.24	0.22	0.05
Uric acid (mg/dl)	2.9 – 7.0	5.89	6.75	7.04	6.5	9.6	7.2	10.0	4.64	6.34	3.53

Supplementary Table B. Full protein names for the abbreviations in Figure 3.

1. Extracellular

Abbreviation	Protein name	Gene Symbol
**Alpha 1-antitrypsin**	Alpha-1-antitrypsin	SERPINA1
**APCS**	Serum amyloid P-component	APCS
**APOA1**	Apolipoprotein A-I	APOA1
**CRP**	C-reactive protein	CRP
**Fibrinogen gamma**	Fibrinogen gamma chain	FGG
**HPRG**	Histidine-rich glycoprotein	HRG
**IGHD**	Ig delta chain C region	IGHD
**IGHM**	Ig mu chain C region	IGHM
**PLTP**	Phospholipid transfer protein	PLTP

2. Membrane

Abbreviation	Protein name	Gene Symbol
**ABCA1**	ATP-binding cassette sub-family A member 1	ABCA1
**Cholesterol plasma membrane**	Cholesterol	—
**Fc gamma RI**	High affinity immunoglobulin gamma Fc receptor I	FCGR1A
**Fc gamma RIII beta**	Low affinity immunoglobulin gamma Fc region receptor III-B	FCGR3B
**Phospholipid plasma membrane**	Phospholipid	—

3. Cytoplasm

Abbreviation	Protein name	Gene Symbol
**ERK1/2**	Mitogen-activated protein kinase 1	MAPK1
**LIPC (HL)**	Hepatic triacylglycerol lipase	LIPC
**LIPE**	Endothelial lipase	LIPG

4. Nucleus

Abbreviation	Protein name	Gene Symbol
**AP-1**	Activator protein 1	JUN
**ATF-2**	Cyclic AMP-dependent transcription factor ATF-2	ATF2
**C/EBPbeta**	CCAAT/enhancer-binding protein beta	CEBPB
**c-Jun**	Transcription factor AP-1	JUN

**CREB-H**	Cyclic AMP-responsive element-binding protein 3-like protein 3	CREB3L3
**HNF1-alpha**	Hepatocyte nuclear factor 1-alpha	HNF1A
**HNF4-alpha**	Hepatocyte nuclear factor 4-alpha	HNF4A
**STAT3**	Signal transducer and activator of transcription 3	STAT3
**TGIF**	Homeobox protein TGIF1	TGIF1

Supplementary Figure A. MetaCore Legend. A legend for the network created in MetaCore suite shown in the Figures 3.

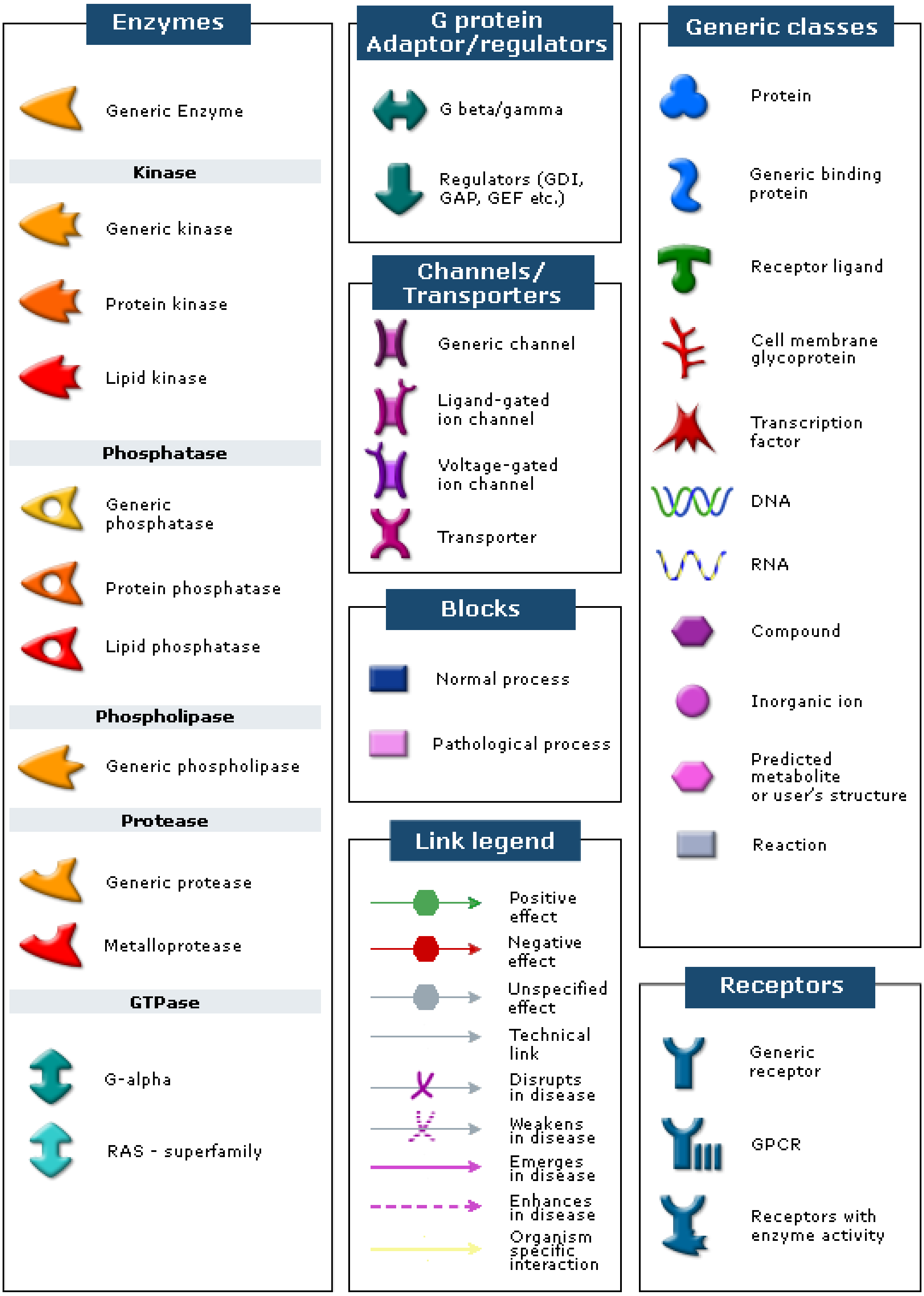

.
